# Long-range recruitment of Martinotti cells causes surround suppression and promotes saliency in an attractor network model

**DOI:** 10.3389/fncir.2015.00060

**Published:** 2015-10-14

**Authors:** Pradeep Krishnamurthy, Gilad Silberberg, Anders Lansner

**Affiliations:** ^1^Department of Numerical Analysis and Computer Science, Stockholm UniversityStockholm, Sweden; ^2^Department of Computational Biology, School of Computer Science and Communication, Royal Institute of Technology (KTH)Stockholm, Sweden; ^3^Department of Neuroscience, Karolinska InstitutetStockholm, Sweden

**Keywords:** Martinotti cells, attractor network, disinhibition, inhibitory interneurons, long-range inhibition

## Abstract

Although the importance of long-range connections for cortical information processing has been acknowledged for a long time, most studies focused on the long-range interactions between excitatory cortical neurons. Inhibitory interneurons play an important role in cortical computation and have thus far been studied mainly with respect to their local synaptic interactions within the cortical microcircuitry. A recent study showed that long-range excitatory connections onto Martinotti cells (MC) mediate surround suppression. Here we have extended our previously reported attractor network of pyramidal cells (PC) and MC by introducing long-range connections targeting MC. We have demonstrated how the network with Martinotti cell-mediated long-range inhibition gives rise to surround suppression and also promotes saliency of locations at which simple non-uniformities in the stimulus field are introduced. Furthermore, our analysis suggests that the presynaptic dynamics of MC is only ancillary to its orientation tuning property in enabling the network with saliency detection. Lastly, we have also implemented a disinhibitory pathway mediated by another interneuron type (VIP interneurons), which inhibits MC and abolishes surround suppression.

## Introduction

Long-range connections in the cortex are ubiquitous and they play a very important role in integrating information originating outside the classical receptive fields (Das and Gilbert, 1995; Boucsein et al., 2011). There are numerous evidences showing that at the level of primary sensory areas, these long-range connections could favor like-to-like features in excitatory neurons (Rochefort et al., 2009; Muir et al., 2011). Recent developments in tracer-injection studies have shed some light upon the fraction of long-range projections on the pyramidal cells (PC) which turned out to be much higher than previously estimated (Stepanyants et al., 2009). Characterization of the physiological properties of long-range projecting synapses have also shown high reliability of these connections (Nawrot et al., 2009). Despite such developments, the cell-type specific innervation patterns of long-range connections still remains unclear (Budd and Kisvárday, 2012). Since the number of excitatory neurons in the cortex outnumbers inhibitory neurons by roughly 4:1 (Markram et al., 2004), excitatory neurons has been viewed as the primary targets of long-range connections (McGuire et al., 1991).

Although excitatory neurons are the principal output neurons, inhibitory neurons have received plenty of attention due to their (1) morphological and synaptic diversity; (2) specific targeting; and (3) ability to directly control the output of principal neurons (Ascoli et al., 2008; Burkhalter, 2008; Hangya et al., 2014). Particularly, Martinotti cells (MC) that receive facilitating synapses from PC have been shown to act as burst detectors and to control the firing rate of PC in a frequency dependent manner (Kapfer et al., 2007; Silberberg and Markram, 2007; Berger et al., 2010). MC have been shown to be involved in a disinhibitory circuit mediated by VIP interneurons (Pfeffer et al., 2013; Pi et al., 2013; Fu et al., 2014) and are also recruited at specific behavioral epochs (Kvitsiani et al., 2013). A recent finding demonstrated preferential recruitment of MC by long-range excitatory projections in the superficial layer of the mouse visual cortex pointing to the possible role of MC in surround suppression and stimulus size tuning (Adesnik et al., 2012). With the prevalence of long-range connections in the cortex, quantitative and qualitative knowledge of long-range projections received by inhibitory neurons would be important given the role of inhibitory neurons in shaping cortical dynamics (Harris and Mrsic-Flogel, 2013).

The objective of this study is to better understand the role played by the long-range inhibition mediated by MC in a cortical attractor network model (Lundqvist et al., 2010; Krishnamurthy et al., 2012; Herman et al., 2013). Many cortical network models, including our own (Krishnamurthy et al., 2012), have hitherto allowed intercolumnar connections only between excitatory neurons (Rolls, 2007). In this work we have relaxed those constraints by letting long-range excitatory projections target both excitatory (pyramidal) and inhibitory (Martinotti) neurons. We find that in the presence of MC-mediated long-range inhibition, the activity level of the network decreased as the size of the stimulus field increased, a phenomenon called surround suppression. We have demonstrated how a network employing this mechanism can make the center salient by evincing a stronger response in the center when there is a contrast between the center and the surround, as reported in experiments (Knierim and van Essen, 1992; Lamme, 1995; Zipser et al., 1996; Kastner et al., 1997). Our results suggest that the synaptic dynamics of the interneurons, depressing or facilitating, plays an important yet a minor role in comparison to its orientation tuning in computing center-saliency, which is the first step in the construction of the bottom-up saliency maps (Li, 2002). We have also implemented a disinhibitory pathway involving MC (Pfeffer et al., 2013), which is activated during locomotion (Fu et al., 2014; Poorthuis et al., 2014) and results in the disappearance of surround suppression as reported recently (Ayaz et al., 2013).

## Materials and Methods

### Model Neurons and Synapses

The cells included were layer 2/3 PC and three different types of inhibitory interneurons. They were parvalbumin-expressing basket cells (BC), somatostatin-expressing MC and vasoactive intestinal polypeptide (VIP) containing cells (Kawaguchi and Kubota, 1996; Douglas and Martin, 2004; Kapfer et al., 2007; Silberberg and Markram, 2007). In the real cortex, BC target the soma of PC and vertically projecting MC innervates the dendritic tufts of PC (Ascoli et al., 2008). This distinction was not captured in our model since our model cells were single-compartmental types. Instead, the distinction between BC and MC was based on their presynaptic mechanisms (see below). Multiple *in vivo* studies have reported VIP-expressing interneurons preferentially inhibiting MC but the identity of VIP containing cells are not clear (Lee et al., 2013; Pfeffer et al., 2013; Fu et al., 2014).

The single-compartment model cells were based upon the Hodgkin-Huxley formalism with size of each cell type’s soma, steady-state current and voltage equations, and conductance values taken from Pospischil et al. (2008; Table 1). The PC were of a regular firing type. Adaptation was modeled using the M-current, which is a slow non-inactivating potassium current. The BC were modeled as non-adapting, relatively fast-spiking cells. The MC and VIP were of the same size as BC except they were both adapting.

**Table 1:** **Neuron parameters**.

Parameter	Pyramidal	Basket	Martinotti	Unit
E_leak_	−70	−70	−70	mV
E_Na_	50	50	50	mV
E_K_	−100	−100	−100	mV
g_leak_	0.0001	0.00015	0.00015	S/cm^2^
g_Na_	0.05	0.05	0.05	S/cm^2^
g_K_	0.005	0.01	0.01	S/cm^2^
g_M_	7e−5	0.000098	0.0001	S/cm^2^
Soma diameter	96	67	67	μm
c_m_	1	1	1	μF/cm^2^

*Single compartment Hogdkin-Huxley model parameters for different classes of cortical neurons taken from Pospischil et al. (2008)*.

All models described here were single-compartment neurons (cylinder of diameter d and length L) described by the following membrane equation:

$$C_{m}\frac{dV}{dt}\;=\;-G_{\text{leak}}(V-E_{\text{leak}})-I_{Na}-I_{K}-I_{M}$$

where, *V* = membrane potential, C_*m*_ = specific capacitance of the membrane, *G*_leak_ = specific (leak) membrane conductance, *E*_leak_ = resting membrane reversal potential. The kinetic parameters of the voltage-dependent *Na* current were given by

$$\begin{array}{l} I_{Na}=g_{Na}m^{3}h(V-E_{\text{Na}})\\ \frac{dm}{dt}=\alpha_{m}(V)(1-m)-\beta_{m}(V)m\\ \frac{dh}{dt}=\alpha_{h}(V)(1-h)-\beta_{h}(V)h\\ \alpha_{m}=\frac{-0.32(V-V_{T}-13)}{\exp[-(V-V_{T}-13)/4]-1}\\ \beta_{m}=\frac{0.28(V-V_{T}-40)}{\exp[(V-V_{T}-40)/5]-1}\\ \alpha_{h}=0.128\exp[-(V-V_{T}-17)/18]\\ \beta_{h}=\frac{4}{1+\exp[-(V-V_{T}-40)/5]} \end{array}$$

where *g*_Na_ and *E*_Na_ of different cortical cells are given in Table 1.

The kinetic parameters of the voltage-dependent K (delayed rectifier) current was given by

$$\begin{array}{l} I_{Kd}=g_{Kd}n^{4}(V-E_{K})\\ \frac{dn}{dt}=\alpha_{n}(V)(1-n)-\beta_{n}(V)n\\ \alpha_{n}=\frac{-0.032(V-V_{T}-15)}{\exp[-(V-V_{T}-15)/5]-1}\\ \beta_{n}=0.5\exp[-(V-V_{T}-10)/40] \end{array}$$

where *g*_Kd_ and *E*_Kd_ of different cortical cells are given in Table 1.

The kinetic parameters of the voltage-dependent M current was given by

$$\begin{array}{l} I_{M}\;=\;g_{M}p(V\;-\;E_{K})\\ \frac{dp}{dt}=(p_{\infty }(V)-p)/\tau_{p}(V)\\ p_{\infty }(V)=\frac{1}{1+\exp[-(V+35)/10]}\\ \tau_{p}(V)=\frac{\tau_{\max}}{3.3\exp[(V+35)/20]+\exp[-(v+35)/20]} \end{array}$$

where *g*_M_ and τ_max_ of different cortical cells are given in Table 1.

Glutamatergic synapses acted on two broad categories of receptors, i.e., kainate/AMPA and NMDA types. A mix of both provided the PC-PC_LO_ and PC-PC_LR_ glutamatergic transmission^1^. It is inconclusive from experiments whether PC-BC, PC-MC_LO_ and PC-MC_LR_ glutamatergic tranmission are plainly kainate/AMPA or a mix (Connors and Cruikshank, 2007; Silberberg and Markram, 2007). A recent study implicated disruption of NMDA receptors specifically in fast-spiking basket cell in cognitive impairments (Carlén et al., 2012). But for simulations presented in this paper, PC-BC, PC-MC_LO_ and PC-MC_LR_ glutamergic transmission were entirely mediated by AMPA. The GABA-ergic transmission in our model found on BC-PC, MC-PC and VIP-MC connections were exerted solely by GABA_A_ (Table 2). AMPA and GABA_A_ currents were given by Destexhe et al. (1994):

$$I_{\text{syn}}\;=\;G_{\text{syn}}s\;(E_{\text{syn}}-V)$$

**Table 2:** **Synapse parameters**.

Pre-Post	Type	EPSP/IPSP amplitude (mV)	Connection probability	Rise time (s)	Delay time (s)	E_rev_(mV)
PC-PC_LO_	Kainate/AMPA	1.2	25%	0.05	0.006	0
PC-PC_LO_	NMDA	0.6	25%	0.005	0.150	0
*PC-PC_LR_	Kainite/AMPA	0.3	−	0.05	0.006	0
*PC-PC_LR_	NMDA	0.15	−	0.005	0.150	0
PC-BC	Kainate/AMPA	1.7	70%	0.05	0.006	0
PC-MC_LO_	Kainate/AMPA	0.1	30%	0.05	0.006	0
*PC-MC_LR_	Kainate/AMPA	0.1	−	0.05	0.006	0
BC-PC	GABA_A_	−1.4	70%	0.05	0.006	−75
MC-PC	GABA_A_	−0.6	80%	0.05	0.006	−75
VIP-MC	GABA_A_	−0.2	60%	0.05	0.006	−75

*There is only a 50% probability that any two minicolumns are connected with the above mentioned connection strength.

where the gating variable *s* (the fraction of open channels) was described by first-order kinetics via two equations:

$$\begin{array}{l} \frac{dx}{dt}=\alpha_{x}\sum_{j}\delta (t-t_{j})-\frac{x}{\tau_{x}}\\ \frac{ds}{dt}=\alpha_{s}x(1-s)-\frac{s}{\tau_{s}} \end{array}$$

The NMDA current was given by:

$$I_{\text{syn}}\;=\;G_{\text{syn}}s(E_{\text{syn}}-V)/(1+[Mg^{2+}]\exp(-0.062V_{m})/3.57)$$

The gating variable s obeyed the same equations as above. We have used τ_x_ = 0.05 ms and τ_s_ = 6 ms for AMPA and GABA_A_, τ_x_ = 5 ms and τ_s_ = 150 ms for NMDA, α_x_ = 1 (dimensionless) and α_s_ = 1(1/ms) for AMPA, NMDA and GABA_A_.

#### Synaptic Short-Term Plasticity

Short-term depression and facilitation were incorporated into all glutamatergic and GABAergic synapses as before (Abbott et al., 1997; Tsodyks et al., 1998; Krishnamurthy et al., 2012). Every presynaptic spike occurring at *t_sp_*, caused a fraction *U* of the available pool to be utilized and the rate of return of resources given by τ_rec_ is multiplied by a quantity *R* (the fraction of available vesicles). *R* obeyed the dynamical equation (Fuhrmann et al., 2002):

$$\frac{dR}{dt}\;=\;\frac{\left(1-R\right)}{\tau_{\text{rec}}}-UR\delta (t-t_{sp})$$

The short-term depression was introduced into the synapse model by multiplying α_x_, which mimics the transmitter release per spike, by *R*, which was the fraction of available vesicles.

In modeling a facilitating synapse, *U* became a dynamic variable, increased at each presynaptic spike and decayed to the baseline level in the absence of spikes.

$$\frac{dU}{dt}\;=\;-\frac{U}{\tau_{\text{facil}}}+U1(1-U)\delta (t-t_{sp})$$

Where *U*1 was a constant that determined the step increase in *U* and τ_facil_ was the decay time constant of facilitation.

The effect of a synapse is strongly depressing if both *U* and τ_rec_ are large and strongly facilitating if *U* is small and τ_facil_ is large. The values assigned for each connections type taken from our previous work can be seen in Table 3 (Gupta et al., 2000; Silberberg and Markram, 2007; Krishnamurthy et al., 2012). The new connections were PC-MC_LR_ and VIP-MC and the dynamics of those synapses were facilitating and depressing respectively.

**Table 3:** **Short-term plasticity parameters**.

Pre-Post	Type	*U*	*U1*	τ_rec_	τ_facil_
PC-PC_LO_	Depressing	0.4	−	600	0
PC-PC_LR_	Depressing	0.4	−	600	0
PC-BC	Facilitating	0.5	−	600	0
PC-MC_LO_	Facilitating	–	0.05	20	1000
PC-MC_LR_	Facilitating	–	0.05	20	1000
BC-PC	Depressing	0.25	−	500	0
MC-PC	Depressing	0.25	−	500	0
VIP-MC	Depressing	0.25	−	500	0

### Architecture of the Network Model

**Figure 1:**
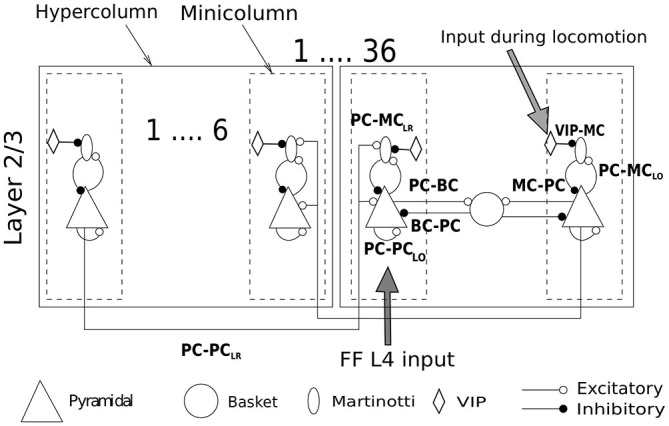
**Network structure and connectivity**. Cartoon of a network of 36 hypercolumns with 6 minicolmns each, showing all the excitatory and inhibitory pathways between cell types within and between columns. See text for more description and Table 2 for connection densities and connection strengths of all the pathways. Two sources of input enter the network. One is the feedforward (FF) layer 4 input carrying the stimulus information. The other is the selective drive to VIP cells during locomotion. The stimulus to VIP remains switched off unless otherwise mentioned.

The sub-sampled neocortical model used here represented a 3 × 3 mm patch of cortex arranged on a square topology of 6 × 6 hypercolumns each with a diameter of 500 μm (Mountcastle, 1997). Each hypercolumn further constituted several minicolumns that served as feature detectors (e.g., orientation, color or motion) in primary sensory areas—various estimates suggest that there are about one hundred minicolumns bundled into a hypercolumn (Peters and Sethares, 1991). Although the presence of anatomical minicolumns in some species (e.g., mouse cortex) are not as clear as in primates (Buxhoeveden and Casanova, 2002; Schenker et al., 2008), data shows the existence of functional subnetworks also in mouse visual cortex and preferential connectivity between cells with similar responses to sensory stimuli (Ko et al., 2011, 2014). In the current sub-sampled network model we had six minicolumns per hypercolumn [a detailed description of our full-scale conceptual model can be found in Lundqvist et al. (2006), the latest developments can be found elsewhere (Lundqvist et al., 2011; Meli and Lansner, 2013; Kaplan and Lansner, 2014) along with our previous work on MC (Krishnamurthy et al., 2012)]. The arrangement of cells in the local microcircuit together with connection probabilities and strengths (PSP amplitudes) are shown in Figure 1. Each minicolumn consisted of thirty PC, three MC and three VIP cells. PC in each minicolumn are connected to 25% of other PC in the same minicolumn (PC-PC_LO_). MC receives input from 40% of PC in the same minicolumn (PC-MC_LO_) and in turn densely innervates 80% of them (MC-PC). Each hypercolumn had 8 BC and BC inhibition that projected laterally to all minicolumns within a hypercolumn is equivalent to non-specific lateral inhibition of BC found in cat (Kisvárday et al., 1994). Thus, each PC targeted 70% of BC in the same hypercolumn and each BC, reciprocally, targeted 70% of PC in the same hypercolumn (Thomson et al., 2002; Douglas and Martin, 2004).

All the synaptic strengths and connectivity between PC and MC followed from our previous modeling work on MC (Krishnamurthy et al., 2012) with the following modifications: (1) Long-range excitatory connections to MC; (2) Orientation specific tuning of MC; and (3) a disinhibitory circuit involving VIP cells. The minicolumns distributed over different hypercolumns that encoded correlated features are connected by long-range connections (PC-PC_LR_). The long-range connections in our earlier networks were only established between excitatory cells in different minicolumns. This is because most of the literature on long-range connections has thus far shown excitatory neurons to be the major recipient of these connections. However, based upon the latest findings (Adesnik et al., 2012), we included long-range connections to MC as well (PC-MC_LR_). In our model, each PC in a minicolumn innervated 4 PC and 1 MC in randomly chosen target minicolumns (or terminal fields) and we assumed one terminal field per PC for all simulations (Voges et al., 2010). Hence, there is a 50% probability that any two minicolumns are connected with the connection strength shown in Table 2. Since there is no direct evidence of long-range connections to BC, long-range connections in our model targeted only PC and MC, unless otherwise mentioned. These are long-range projections spanning within an area, primarily within the primary sensory areas, and are different from long-range connections that span multiple areas. Furthermore, since MC have been shown to be orientation-selective (Ma et al., 2010), the extent of MC inhibition in our model was limited to a minicolumn. BC on the other hand, were orientation non-selective and thus provided common inhibition to all minicolumns in a hypercolumn like in our previous works. Recent studies on VIP Cre-recombinase driver mouse line (Taniguchi et al., 2011) have shown long-range excitatory projections from motor cortex evoking the strongest response in VIP cells with somatostatin-expressing interneurons receiving the strongest inhibition from photoactivated VIP cells (Lee et al., 2013; see “Discussion” Section for the behavioral consequences of the VIP disinhibition). This is also consistent with reports from other labs (Fu et al., 2014). This has been incorporated in our model by the introduction of VIP-MC connection.

Each model neuron in the network was assigned a three-dimensional (*x*, *y*, *z*) coordinate and all conduction delays were calculated assuming a mean conduction speed of 0.3 m/s (unless otherwise stated). The Gaussian distribution of conduction speed (width controlled by standard deviation) and the distribution of cell-to-cell distance gave rise to the gamma-family distribution of delays. The model was built using the NEURON simulator (Hines and Carnevale, 1997) and the simulations were typically performed on 144 cores of the Cray XE6 supercomputer at the Center for High Performance Computing (PDC) at KTH.

### Input to Minicolumns and VIP clls

We used a point-conductance model of synaptic noise to account for the stochastic variation of conductance due to synaptic background activity on all cell models (Destexhe et al., 2001). We used the parameter mean conductance (*g_noise_* = 0.000121 μS) to achieve a low firing rate background noise (0.5–1 Hz) in PC mediated by AMPA synapses. The same mechanism was also used to provide input to the network. Independent spike trains generated for the duration of 1 s targeted PC in each minicolumn. The number of minicolumns getting direct activation is described in each stimulus condition with the strength of the stimulus modulated by the mean conductance (*g*_i_). The following are the different levels of external stimulatuon (*g*_1_, *g*_2_, *g*_3_, *g*_4_, *g*_5_) = (0.0028, 0.0024, 0.0020, 0.0022, 0.0025) μS and *g*_2_ is the default input unless otherwise mentioned. Since our network model represents cortical layer 2/3, the input to each minicolumn was akin to feedforward (FF) cortical layer 4 input and they are private to each minicolumn. Although we did not use a special input cortical layer as in our previous works, the implementation of minicolumnar activation during different stimulus conditions is quite similar to our previous works (Lundqvist et al., 2006, 2010, 2011). We have assumed selective drive to VIP cells during disinhibition with the strength of the stimulus drive modulated by the mean conductance (*g*_input_inh_ = 0.0018 μS).

## Results

Our network model with hypercolumnar and minicolumnar structure representing a cortical patch covering an area of 3 × 3mm^2^ consisted of single-compartment Hodgkin-Huxley type model neurons with short-term plasticity included in all their synapses. The model consisted of 36 hypercolumns with six minicolumns in each hypercolumn. PC in the same minicolumn were recurrently connected (PC-PC_LO_) and PC in minicolumns distributed across different hypercolumns that encoded correlated features were connected by long-range connections (PC-PC_LR_). The synaptic dynamics of both PC-PC_LO_ and PC-PC_LR_ were depressing. The network model contained three interneuronal populations—BC, MC and VIP. BC were reciprocally connected to PC through depressing synapses (PC-BC and BC-PC) and each hypercolumn acted as a winner-take-all (WTA) module due to common inhibition provided by BC. MC, on the other hand, received facilitating synapses from PC (PC-MC_LO_ and PC-MC_LR_) and connected to PC through depressing synapses (MC-PC). The facilitating nature of PC-MC_LO_ synapses helped to regulate the activity level of PC by exerting frequency dependent disynaptic inhibition on PC (Silberberg and Markram, 2007; Krishnamurthy et al., 2012). Inhibition of MCs was performed by VIP interneurons, which preferentially inhibited MC via depressing synapses.

Previously, we showed that the spontaneous activity of our attractor network in the absence of an external stimulus hopped between different attractor states (Krishnamurthy et al., 2012). The amount of time the network spent in an attractor was reported to be controlled by synaptic depression, cellular adaptation and MC firing. We also demonstrated that MC with their characteristic facilitating synapses from PC played a dominant role in attractor termination. In the current study we subjected our network to an external stimulation since one of the objectives of this work was to investigate the effect of intercolumnar inhibition (PC-MC_LR_) mediated by MC during presentation of stimuli with different sizes. External stimulation was delivered to PC in a minicolumn and hence stimulation of any of the stored attractor either partially or fully was accompanied by elevated firing of PC belonging to that attractor followed by BC and MC firing. Our main focus here has been to compare the responses of the network with and without PC-MC_LR_ (see “Materials and Methods” Section for more information on the structural changes to our previous network model). The impact of VIP disinhibition was not taken up until see “VIP-Mediated Cortical Disinhibition” Section, hence these cells remained inactive in the model until then.

### Long-Range Inhibition Causes Surround Suppression

**Figure 2:**
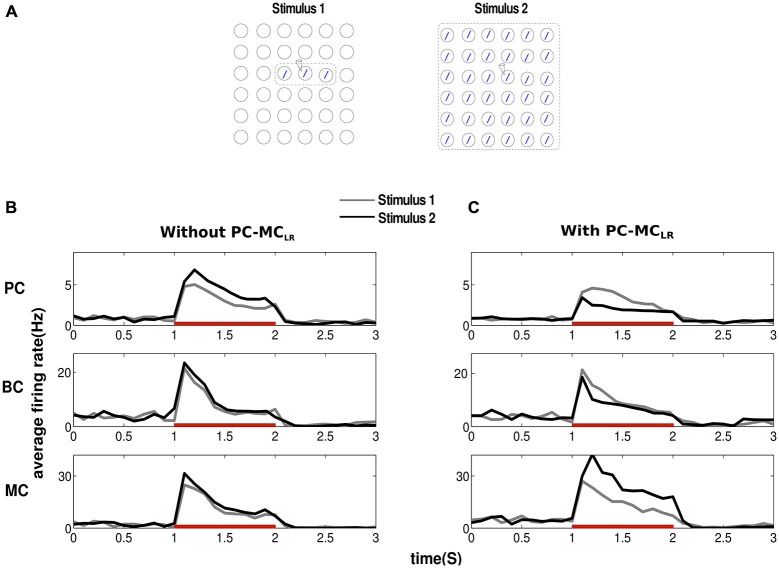
**Long-range disynaptic inhibition causes surround suppression. (A)** To study surround suppression, we chose stimuli of two different sizes and magnitude *g_2_* (see “Materials and Methods” Section) electrode symbol denotes the minicolumn from where activity was recorded. The results obtained were averages from 10 trials.** (B)** Before the arrival of the stimulus, the network is at low baseline firing rate. Since the recorded minicolumn was part of the external stimulation, average firing rate of pyramidal cells (PC) show elevated firing after stimulus onset. Red bar represents the period of stimulus presentation. In our traditional network without long-range disynaptic inhibition (PC-MC_LR_), the average firing rate of PC increased with the size of the stimulus failing to demonstrate any surround suppression. Basket cells (BC) and martinotti cells (MC) firing rate was similar to PC showing lack of surround suppression. **(C)** Introducing PC-MC_LR_ in the network resulted in marked surround suppression in PC and BC. MC activity shows a complete lack of surround suppression with its firing rate going up with the size of stimulus.

In order to study surround suppression, we applied stimuli of two different sizes to our networks activating the same pattern (Figure 2A). Both stimuli (Stimulus 1, Stimulus 2) activated the same pattern (one minicolumn per hypercolumn but in different hypercolumns) in different proportions. While Stimulus 1 activated only three minicolumns of the pattern, Stimulus 2 activated all the minicolumns, i.e., the area of stimulation covered by Stimulus 2 was larger than Stimulus 1. To calculate the mean firing rate of a minicolumn, we chose a random minicolumn (denoted by an electrode symbol in the Figure 2A) from the set of minicolumns that received direct stimulation. We averaged the activity of all the cells from the chosen minicolumn with a bin size of 100 ms. The result of the application of this input protocol to the network without PC-MC_LR_ is shown in Figure 2B. In agreement with our previous network models with long-range connections solely between PC in different hypercolumns (PC-PC_LR_), the mean firing rate of PC increased with the size of the stimulus, i.e., they failed to demonstrate surround suppression (Figure 2B, top panel). Furthermore, during the presentation of both stimulus configurations, the firing rate of PC initially increased after the input onset until the effect of cellular adaptation and synaptic depression became strong enough to bring the firing rate down. Similar to PC, the average activity of BC (Figure 2B, middle panel) and MC (Figure 2B, bottom panel) also failed to demonstrate surround suppression when the size of the stimulus got larger.

When PC-MC_LR_ were introduced, the increase in the stimulus size resulted in an increased excitatory drive to MC through the recruitment of long-range connections by PC outside their hypercolumn. This led to an increase in the firing rate of MC (Figure 2C, bottom panel) accompanied by a reduction in the average activity of PC (Figure 2C, top panel) and BC (Figure 2C, middle panel). These results suggest that long-range inhibition mediated via MC could implement surround suppression as the size of the stimulus field got larger. Hence, the activity of PC and BC in our modified network demonstrated surround suppression while the activity of MC displayed lack thereof in agreement with the experimental data (Adesnik et al., 2012).

### Contextual Interactions in the Presence of Long-Range Inhibition

**Figure 3:**
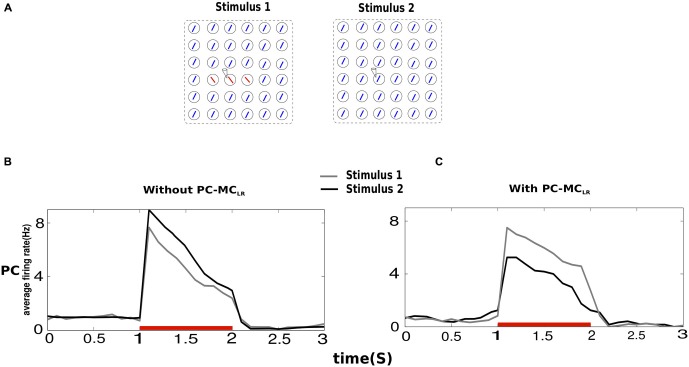
**Contextual interactions in the presence of long-range disynaptic inhibition. (A)** In Stimulus 2, the recorded hypercolumn was part of the homogeneous background and in Stimulus 1, the recorded hypercolumn was in a contrasting background. The results obtained were averages from 10 trials and both Stimulus 1 and Stimulus 2 were of magnitude *g_2_* (see “Materials and Methods” Section). **(B)** The average firing rate of PC indicate that in a network without PC-MC_LR_, the saliency of a bar in a homogeneous texture is larger than when it is in a contrasting background shown by the average activity of the former larger than the latter. **(C)** In the presence of PC-MC_LR_, a bar present in a contrasting background was more salient.

The response of PC to a stimulus in its receptive field has been shown to be context dependent. Surround suppression (demonstrated above) and contour enhancement are some examples of physiologically observed phenomena of contextual influence (Knierim and van Essen, 1992; Kapadia et al., 1995). Hence, we studied the effects of PC-MC_LR_ when we introduced small changes in the stimulus field. Both input configurations (Stimulus 1, Stimulus 2) were applied to the entire network (Figure 3A). In Stimulus 2, the recorded minicolumn was part of the homogeneous background but in the case of Stimulus 1, a contrasting background surrounded the center.

In the absence of PC-MC_LR_, the mean firing rate of PC during Stimulus 2 was larger than Stimulus 1. This is because during Stimulus 2, the activity of PC in the iso-orientation minicolumns in other hypercolumns enhanced the activity of PC in the recorded minicolumn, which in not the case during Stimulus 1 because the activity of PC in the recorded minicolumn was unaided by contrasting background. When PC-MC_LR_ were switched on, the behavior of the network turned around. This is because MC in the recorded minicolumn during Stimulus 2 emitted more spikes than MC in the recorded minicolumn during Stimulus 1 leading to stronger suppression of PC in the recorded minicolumn during Stimulus 2. Hence, in the presence of MC-mediated long-range inhibition, the network response to the center with contrasting surround was larger than the one with homogeneous texture.

### The Response of Different Network Structures to Center-Surround Stimulus

**Figure 4:**
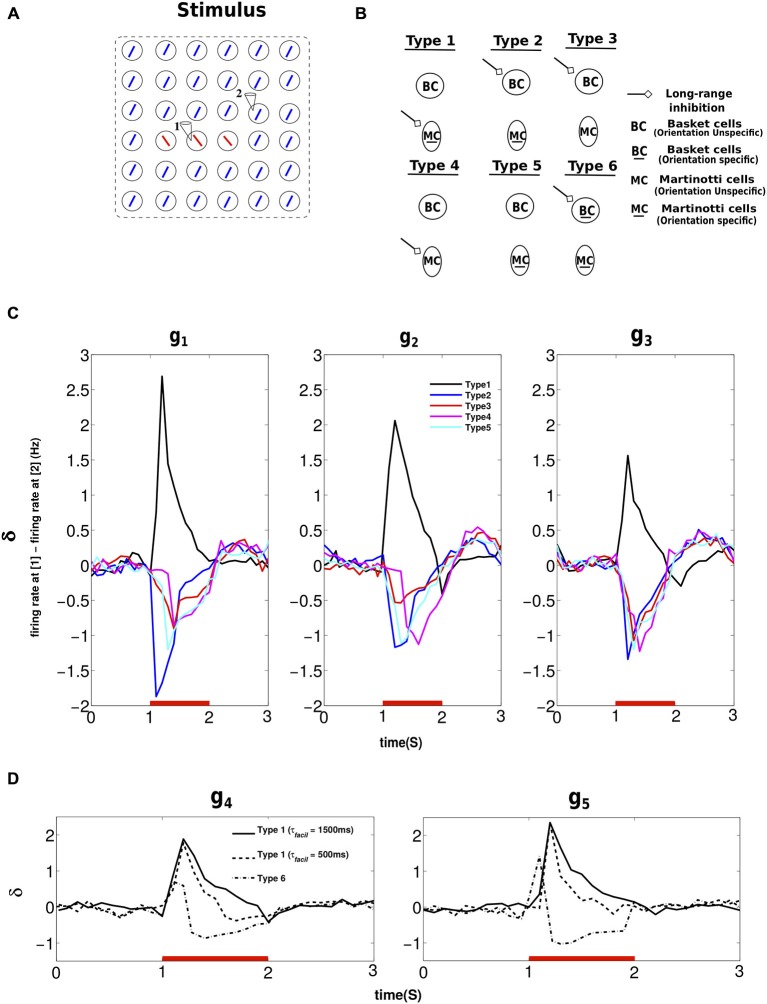
**Response of different network structures to center-surround stimulus configuration. (A)** The stimulus activated the entire patch as shown. The hypercolumn at recording site “1” is part of the center and the hypercolumn at site “2” is part of the surrounding texture. *δ* refers to the difference in the firing rates at “1” and “2”. The results obtained were averages from 10 trials.** (B)** Cartoon depicting connection schemes employed to demonstrate the effect of center-surround stimulus configuration on different network structures. The arrow denotes long-range inhibition mediated by BC or MC when they assume extreme values of orientation specificity.** (C)** We used stimuli of three different strengths (*g_1,_ g_2,_ g_3_*) for this experiment and hence the three panels. The red bar denotes the period of stimulus presentation and plotted are the difference in the average activity of PC at “1” and “2” when the stimulus was presented to different network structures. When the inhibition was mediated by orientation unspecific inhibitory neurons, regardless of their presynaptic dynamics, that is depressing (BC) or facilitating (MC), the global inhibition to the minicolumn at the center was less than the surround (see text for more description). Hence, this resulted in negative *δ* for Type 2, Type 3, Type 4 and Type 5 network structure. On the contrary, *δ* was positive only for the network structure with long-range inhibition inhibition mediated via orientation specific MC (Type 1).** (D)** For this experiment, we used stimuli of two different strenghts (*g*_4_, *g*_5_) and hence the two panels. Here again *δ* refers to the difference in the firing rates at “1” and “2”. Lowering τ_facil_ of PC-MC_LO_ and PC-MC_LR_ connections lowers the duration of postive *δ*. When long-range inhibition was mediated by orientation specific BC (Type 6), the duration of positive *δ* furthur deteriorated. The initial positive *δ* was followed by a negative *δ* due to the depressing synapses between PC and BC (see text).

The above results indicated the enhancement of activity in the center relative to the surround owing to MC mediated long-range inhibition. Next, we studied how the network response to the center was affected by: (1) changing the orientation tuning property of MC; and (2) switching from MC- to BC-mediated long-range inhibition (Figure 4B). When we changed the orientation tuning of MC, from finely tuned (Type 1) to broadly tuned (Type 4), the MC subpopulations in different minicolumns in each hypercolumn merged to form a common inhibitory pool just like BC. This means the number of MC per hypercolumn is more when it is orientation selective. The increase in the number of MC per hypercolumn is compensated so that the product (number of MC neurons per hypercolumn * number of incoming projections * weight per connection) is kept constant. This type of compensation was also implemented when long-range connections to MC were removed (Type 5). Similarly, connection weights between PC and BC were adjusted to compensate for added connections while switching from MC- to BC-mediated long-range inhibition to maintain the overall inhibition in the network (Type 2 and Type 3). Figure 4C shows the results of applying stimuli of three different strengths as sketched in Figure 4A. “1” and “2” mark the activity recorded at minicolumns in different hypercolumns and *δ* denotes the difference in their firing rates. Positive *δ* indicates a saliency map with the activity in the center higher than the surround and a negative *δ* means a saliency map with the activity in the surround higher than the center.

Type 1 indicates the connection scheme (Figure 4B) employed thus far in sections long-range inhibition causes surround suppression and Contextual interactions in the presence of long-range inhibition, i.e., long-range inhibition mediated by orientation specific MC. MC in the minicolumn that was part of the surround (site “2”) fired more action potentials than MC that belonged to the minicolumn in the center (site “1”). This resulted in stronger suppression of PC in the minicolumn at site “2” and a pronounced positive *δ* as shown in Figure 4C. In the connection scheme without any long-range inhibition (Type 5), *δ* was briefly around zero initially (not visible in the Figure due to a bin size of 100 ms) since the minicolumns both at site “1” and “2” were input driven. Then the activity of PC that belonged to the surround (site “2”) went higher than the other pattern (site “1”). Since all the inhibition is mutually shared in Type 5 connection scheme, the collective action of the surround went higher than the center because the number of active minicolumns in the surround was more numerous than in the center. Thus the average activity of PC at site “1” was overcome by the stronger inhibition exerted by the surround resulting in the average activity at site “1” going below that of site “2” and hence the negative *δ*. Long-range inhibition is mediated by orientation unspecific BC in Type 2 and Type 3 and by orientation unspecific MC in Type 4. As long as the long-range inhibition was exerted by orientation unspecific inhibitory cells (Type 2, Type 3 and Type 4), the network response to input salience was negative and the explanation is similar to the network response of Type 5.

In order to understand the role of short-term synaptic properties of interneurons, we employed the same stimulus protocol to: (1) Two different Type 1 networks with different short-term facilitation time constants, τ_facil_ = (1500 ms, 500 ms), between PC and MC (both PC-MC_LO_ and PC-MC_LR_); and (2) A Type 6 network where long-range inhibition was mediated by orientation specific BC. For this experiment, we applied stimuli of two different strengths. The response of the network with τ_facil_ is shown in Figure 4D, it is similar to the Type 1 response in Figure 4C. *δ* was also positive for τ_facil_ with the same peak amplitude but with a shorter duration which is to be expected. When long-range inhibition was mediated by orientation specific BC, the value of *δ* was positive but the duration of its positive value further deteriorated and also resulted in a reversal of the sign. This is because BC in the minicolumn that was part of the surround elicited more spikes than BC in the center; this resulted in the activity in the center exceeding the surround initially and hence positive *δ*. Since synapses that are more active depress stronger than the less active ones, the initial positive *δ* was followed by the stronger depression of PC-BC synapses in the center than the surround leading to a reversal of the sign of *δ*. This indicated that the network promoted input saliency with positive *δ* when the long-range inhibition was mediated either by orientation specific MC (Type 1) or BC (Type 6). But the amplitude of *δ* was strongest and with prolonged duration only for facilitating synaptic dynamics onto MC. Our results suggest that the synaptic dynamics of the interneurons, depressing or facilitating, plays an important yet a minor role in comparison to its orientation tuning in promoting input saliency, which might explain the orientation selectivity of MC seen in experiments (Ma et al., 2010).

### VIP-Mediated Cortical Disinhibition

VIP-mediated disinhibition of cortical PC has been widely reported in primary visual cortex (Fu et al., 2014), auditory cortex (Letzkus et al., 2011) and in somatosensory cortex (Lee et al., 2013). This circuit motif is activated by cholinergic inputs from basal forebrain (BF) during locomotion, auditory fear learning and whisking by increasing the gain of cortical processing. Besides increasing visual responses (Bennett et al., 2013), locomotion has also been shown to weaken surround suppression (Ayaz et al., 2013). In order to implement cortical disinhibition, we introduced VIP cells (see “Materials and Methods” Section). The inputs to this experiment are stimuli of different sizes. Controlling the size of the stimulus field is equivalent to the activation of minicolumns of the same pattern in different hypercolumns. Furthermore, VIP in the activated minicolumns also received input during locomotion. Whilst the origin of FF inputs to PC is layer 4, the inputs targeting VIP during locomotion is assumed to be from BF (see “Discussion” Section).

**Figure 5:**
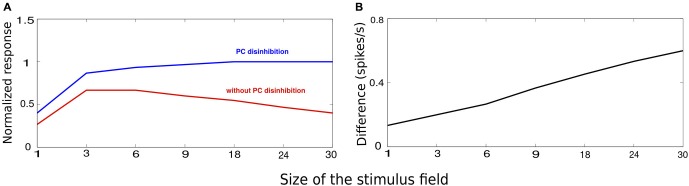
**VIP-mediated disinhibition**. The stimuli chose for this experiement is similar to Figure 2A with the size of the stimulus controlled by the number of activated minicolumns. **(A)** Average minicolumnar response during absence (red) and presence (blue) of VIP-mediated disinhibition normalized to peak response. **(B)** The vertical axis denotes the difference between the responses in absence of presence of VIP-mediated disinhibition. The effect of disinhibition grows with the stimulus size.

Figure 5A shows the average response at the recorded minicolumn when we increased the size of the stimulus. Consistent with the previous reports on monkey (Fitzpatrick, 2000) and mouse (Gao et al., 2010; Adesnik et al., 2012), the average response first increased with the increasing size of the stimulus, followed by a suppression as the stimulus size increased more. The initial upward trend in the response is due to the weak long-range recruiment of MC. This is due to the facilitating nature of PC-MC connections that are endowed with low initial release probability and small unitary EPSPs (Silberberg and Markram, 2007; Krishnamurthy et al., 2012; Table 2). As the stimulus size increased beyond an optimal value, the recruitment of long-range MC got stronger bringing the activity down. This activity is similar to the activity seen during the stationary state (Figure 5A, compare with Figure 2C, red trace in Ayaz et al., 2013). To this network, when we introduced selective drive to VIP cells mimicking locomotion, it abolished surround suppression (Figure 5A, compare with Figure 2C, blue trace in Ayaz et al., 2013). The activation of VIP cells inhibited MC, releasing PC from the clutches of MC inhibition and hence abolished surround suppression. VIP-mediated disinhibtion increased the response to the larger stimuli more than the smaller stimuli and this difference grew linearly with the stimulus size (Figure 5B, compare with Figure 2F in Ayaz et al., 2013).

## Discussion

In order for the brain to process the vast amounts of information reaching the visual system with its limited resources, it has been suggested that the brain constructs saliency maps to devote attention for further processing. In this way features with high saliency could capture gaze preattentively (Treisman and Gelade, 1980). Although the dominant view is that saliency maps are constructed in the global workspace in higher order areas like prefrontal cortex and parietal cortex which then directs attention to a spot with the highest activation (Itti and Koch, 2001; Bisley and Goldberg, 2010), it has been suggested that saliency maps could be constructed even at the level of primary visual cortex. According to the V1 saliency hypothesis (Li, 1999, 2002), there is a direct link between bottom-up saliency maps and feature extraction (e.g., orientation, color or motion) at V1 and it does not require feedback from higher order cortical areas (Hupé et al., 2001). Bottom-up saliency maps, which also underlie perceptual pop-out phenomena is observed whether animals are awake (Knierim and van Essen, 1992) or under anesthesia (Nothdurft et al., 1999). Also, the currency in which saliency is expressed was shown to be the firing rate of cells (Li, 2002; Zhang et al., 2012).

The calculated saliency values are not absolute measures but are relative to the surrounding stimuli. Physiological findings have shown that in layers 2 and 3 of V1 where there are numerous lateral connections, the response of PC depends both on the stimulus in its receptive field and the context. It is higher for an input with a contrasting background than when it is part of a homogeneous texture of orientation bars pointing to the suppressive nature of long-range connections (Knierim and van Essen, 1992; Kapadia et al., 1995). One of the suggested mechanisms to capture iso-orientation suppression is long-range connections targeting both excitatory and inhibitory neurons (Grossberg and Mingolla, 1985; Malik and Perona, 1990; Stemmler et al., 1995). This is not in accordance with most of the literature hitherto on lateral interactions in the cortex (Boucsein et al., 2011) as the evidence for inhibitory neurons receiving long-range projections still remains scant. With the help of optogenetics (Miesenböck, 2009), a recent study demonstrated the role played by MC in surround suppression. The long-range axons from PC were shown to specifically target MC and not BC (Adesnik et al., 2012).

This inspired us to extend our previous cortical attractor network by adding long-range projections to MC (Figure 1). In a network that lacks long-range connections to MC, lateral connections were only facilitatory and this led to an increase in activity as the size of the stimulus got larger. This failed to describe the contextual modulation of cortical responses to visual stimuli (Figure 2). Introducing long-range connections to MC led to the recruitment of MC when the size of the stimulus got larger and as a consequence, the stronger suppression of PC as reported by various experimental studies (Knierim and van Essen, 1992; Lamme, 1995; Zipser et al., 1996; Kastner et al., 1997). It could be interpreted that the presence of an individual orientation bar (also called a singleton) in the visual field is more salient and hence evokes more activity than a homogeneous texture of iso-orientation bars that is less surprising.

The response of our network with MC mediated long-range inhibition was stronger when there was a contrast between the center and the surround compared with the situation of no such contrast (Figure 3; Knierim and van Essen, 1992). This is in agreement with what is proposed by the V1 saliency hypothesis according to which the saliency of a bar, reflected in the firing rate, surrounded by contrasting orientation would be higher than when it is a part of its background (Li, 1999, 2002). The network without MC-mediated long-range inhibition failed to demonstrate this; in fact the saliency map this network gave rise to was quite the opposite. Enhanced activity in the center relative to surround has been suggested as the physiological basis for the psychophysical pop-out effect (Treisman and Gelade, 1980). Human subjects usually detect the presence and identity of target elements embedded in distractor elements effortlessly, regardless of the number of distractors, especially if the target elements differ from the distractor elements in less complicated ways (Bergen and Julesz, 1983) like the stimuli used in this work. It is possible that during pop-out effects, a preattentive system could be constructing bottom-up saliency maps and detecting contrasting features in an image rapidly and draw the window of attention to the region that is more salient for further processing.

In addition, we have shown that the enhanced firing rate at the center relative to surround (*δ*) was positive for both orientation specific BC- or MC-mediated long-range inhibition but the amplitude and the duration of this enhanced relative firing rate was strongest and much prolonged if the synapses between PC and interneurons exhibited facilitating dynamics (Figure 4). Our results indicate that the role of presynaptic short-term plasticity properties of MC in promoting input saliency is only minor to the orientation specificity of MC.

### Relationship to Texture Segregation

Texture segmentation is a task shown to be divided into various subprocesses engaging different cortical layers (Lamme, 1995; Self et al., 2013). The first step is the extraction of orientation of line segments by the FF connections from LGN to V1 (Ferster and Miller, 2000) followed by edge detection between the figure and ground. This is suggested to be achieved in layers 2 and 3 by long-range inhibition (Li, 1999; Roelfsema et al., 2002; Bhatt et al., 2007). Finally, all image elements of the figure are grouped by feedback connections from higher order areas terminating in layers 1, 2 and 5 (Self et al., 2013). Pharmocological disruption of feedback connections from higher areas to V1 is shown not to abolish edge detection (Hupé et al., 2001) as this operation is intrinsic to V1 in agreement with our model and the V1 saliency hypothesis.

### Other Computational Models

All the data on surround suppression so far comes mainly from cats (DeAngelis et al., 1994) and monkeys (Angelucci and Bressloff, 2006). The availability of various genetic tools to target individual neurons makes mouse as a model species more appealing and added to this is the ability to perform experiments in awake-behaving mouse, thereby making it physiologically relevant. Self et al. (2014) recently demonstrated surround suppression in layer 4 of mouse visual cortex, which forms the input to layer 2/3. This means along with horizontal interactions within layer 2/3 (Adesnik et al., 2012), surround suppression could be partly inherited from layer 4 and even from LGN. What is not clear however is how the feedback connections underlie surround suppression in mouse V1. Top-down connections in the cortex are numerous and amongst other layers, they also terminate extensively in the superficial layers of V1 and target both excitatory and inhibitory neurons (Gonchar and Burkhalter, 2003; Anderson and Martin, 2009). Some clues on the contributions of FF, lateral and feedback connections in surround suppression comes from primate V1 (Angelucci and Bressloff, 2006), which has inspired some modeling studies that we will mention very briefly.

Surround suppression is contrast dependent, that is, the radius of the stimulus that evokes peak respone is smaller at high contrast than at low. Somers et al. (1998) built a spiking cortical model to show the contrast modulation of stimulus within classisial receptive field. The model consisted of a grid of minicolumns representing different orientations with each minicolumn compirsing excitatory and inhibitory. Both excitatory and inhibitory neurons made short-range connections while only excitatory connections made long-range connections targeting both excitatory and inhibitory neurons. The main assumption of the model was the asymmetry in the response of excitatory and inhibitory neurons in agreement with the model proposed by Lund et al. (1995). This led to excitatory neurons dominating at very short distance and inhibitory neurons dominating in a local ring around them. The PC-MC facilitating synapses in our model is similar to the excitatory-inhibitory asymmetry of Somers et al. (1998). Hence, a stronger input (modulated by size or intensity) recruits more MC through horizontal connections than a weak input leading to stronger suppression.

Some evidences for the involvement of feedback connections in surround suppression have come from inactivation studies. Cooling area MT in macaque (Hupé et al., 1998) and posterotemporal visual cortex in cat (Bardy et al., 2009) have shown to reduce surround suppression in V1. More evidence comes from recent studies on the spatiotemporal properties of surround suppression that has shown independence of the onset latency of surround suppression to cortical distance (Bair et al., 2003). Surround suppression based on slow conducting horizontal connections cannot explain this satisfactorily. This had led to the suggestion that feedback connections should underlie surround suppression because feedback connections are highly divergent covering a large area and they also conduct at velocities 10 times faster than FF connections. Schwabe et al. (2006) extended their previous model by introducing interareal feedback connections. By employing realistic conduction velocites to horizontal and feedback connections, they demonstrated fast onset and large spatial extent of surround suppression. They also managed to reproduce contrast dependence of suppression strength and timing and dynamics of surround response in agreement with the experiment (Bair et al., 2003).

The role played by long-range inhibition in border detection and constructing saliency maps have been analyzed by previous computational models. In an elegant study by Roelfsema et al. (2002), they used a multi-layered network to model a complete texture segmentation task with the FF pathway detecting boundaries and the feedback pathway handling region filling. Our model has severe limitations as it is only single-layered and long-range connections are not distance-dependent. However, unlike the above mentioned studies that used abstract non-spiking units, our spiking version utilizes dynamic synapses and division of labor among interneuronal subtypes in creating the bottom-up saliency maps.

Another limiation of this study is the use of single-compartment neurons by which we were not able to address how the unique morphological features of MC contributes to surround suppression. MC mainly target the distal dendrites of PC (Silberberg and Markram, 2007), which is the site of dendritic calcium spikes and also where the top-down connections from higher order areas arrive (Larkum et al., 1999). As described above, since surround suppression may involve contributions from horizontal and feedback inputs, it is vital to know the interplay of excitation and inhibition in different domains of PC dendrites, which may shed light upon active dendritic processes involved in this phenomenon (London and Häusser, 2005).

The involvement of somatostatin-expressing interneurons (MC) has been suggested but never been modeled to our knowledge. We predict that silencing MC in V1 using optogenetic tools or selectively obstructing its orientation tuning should severely disrupt bottom-up saliency maps (Cardin et al., 2009).

### Cholinergic Recruitment of Interneurons

The neuromodulator acetylcholine has been shown to mediate functions such as arousal, attention, information gating and learning and memory (Eggermann and Feldmeyer, 2009; Lee and Dan, 2012; Sara and Bouret, 2012; Varela, 2013). The main source of cortical acetylcholine is BF that has shown to be active during alert wakefulness and rapid-eye-movement sleep (Lee and Dan, 2012). Traditional views on the diffuse and non-specific nature of cholinergic projections on cortex has been challenged by recent studies that has demonstrated more structured and topographically organized cortical innervation patterns of cholinergic terminals (Zaborszky, 2002; Zaborszky et al., 2005, 2015). Also, cortical acetylcholine release has been shown to operate on multiple timescales, from milliseconds and tens of milliseconds to minutes and hours (Parikh et al., 2007; Sarter et al., 2009).

The effect of acetylcholine on PC via muscarinic and nicotine receptors has been well documented (McCormick and Prince, 1985; Gil et al., 1997; Kimura and Baughman, 1997; Disney et al., 2007; Kawai et al., 2007). Since the function of PC is also tightly controlled by interneurons (Burkhalter, 2008; Hangya et al., 2014), the impact of acetylcholine on the activity of interneurons has been receiving wide interest. It is believed that probing into the action of acetylcholine on interneurons will help shed more light upon the rich and complex mechanisms by which acetylcholine modulates the information processing in neocortical circuits (Petersen, 2014). Some *in vitro* studies have recently made some inroads into understanding differential effects of acetylcholine on cortical interneurons. A study performed by Alitto and Dan (2012) on anesthetized animals revealed strong activation of VIP and layer 1 interneurons accompanied by a decrease in the activity of pyramidal neurons and PV interneurons, during BF stimulation. This has also been confirmed by other slice studies in which the optogenetic stimulation of cholinergic fibers led to a wave of inhibition in PC and fast-spiking cells as well as nicotinic-receptor mediated excitation of layer 1 and non fast-spiking layer 2/3 interneurons (Arroyo et al., 2012). Since PV interneurons do not show direct responses to acetylcholine (Kruglikov and Rudy, 2008), one likely interpretation for the reduction of PV and PC activity is inhibition via layer 1 and VIP interneurons.

Although results derived from slices and anesthetized animals helps understanding cell-type specific action of acetylcholine, only experiments during behavior in intact brain will ascertain the underlying mechanisms. VIP inhibition of somatostatin interneurons (SST) is one such mechanism many groups have reported to be present in awake conditions but absent under anesthesia (Letzkus et al., 2011; Lee et al., 2013; Fu et al., 2014). This circuit motif is shown to be recruited in primary visual cortex during locomotion (Fu et al., 2014), in auditory cortex during the presentation of aversive stimuli (Letzkus et al., 2011) and in somatosensory cortex during whisking (Lee et al., 2013). Fu et al. (2014) found locomotion increasing the gain of PC in V1 without altering their orientation selectivity. The majority of neurons that were active during locomotion even in the absence of any visual stimulation were VIP neurons. Consistent with the findings of Pfeffer et al. (2013) that reported VIP neurons mainly innervating SST neurons, Fu et al. (2014) also found a strong inhibition of SST neurons during locomotion.

Locomotion has also been found to weaken surround suppression, which is in full agreement with our results. In our network model, VIP cells get activated during locomotion. The activated VIP cells inhibit MC, a well-established pathway discussed above, and abolishes surround suppression by disinhibiting PC. Ayaz et al. (2013) only speculated and did not test the effect of MC inactivation on the reduction in the strength of surround suppression, which is a key prediction of our model. Now that we have specific transgenic lines targeting particular cell types (Taniguchi et al., 2011), future experiments can directly test this idea by silencing VIP cells and demonstrate their role in surround suppression. Also, we report eradication of surround suppression during MC inactivation as opposed to Ayaz et al. (2013) who report a decrease in the strength of surround suppression. This means that it is possible that there could also be other mechanisms responsible for surround suppression in the cortex even if MC were to play a dominant role. Further complicating the picture is, as discussed above, the complex action of acetylcholine on interneurons.

Even though it is difficult to understand the purpose of the effect locomotion has on surround suppression, this phenomenon must be seen in the context of survival. In the real world, as opposed to a laboratory setting, locomotion is an important behavior that signifies either running towards a prey or running away from a predator, with the amount of cholinergic activation and hence VIP-mediated disinhibition of PC proportional to the speed of locomotion (Fu et al., 2014). This could promote rapid and reliable detection of emotionally salient stimuli in our environment.

## Conflict of Interest Statement

The authors declare that the research was conducted in the absence of any commercial or financial relationships that could be construed as a potential conflict of interest.

## Footnotes

^1^PC-MC_LO_ is local PC projecting to MC, PC-MC_LR_ is distant PC projecting to MC via a long-range connection.
